# Identification of molecular biomarkers and pathways of NSCLC: insights from a systems biomedicine perspective

**DOI:** 10.1186/s43141-021-00134-1

**Published:** 2021-03-19

**Authors:** Rakibul Islam, Liton Ahmed, Bikash Kumar Paul, Kawsar Ahmed, Touhid Bhuiyan, Mohammad Ali Moni

**Affiliations:** 1 Department of Software Engineering, Daffodil International University (DIU), Ashulia, Savar, Dhaka, 1342 Bangladesh; 2 Department of Information and Communication Technology, Mawlana Bhashani Science and Technology University, Santosh, Tangail, 1902 Bangladesh; 3 Group of Bio-photomatiχ, Mawlana Bhashani Science and Technology University (MBSTU), Santosh, Tangail, 1902 Bangladesh; 4 WHO Collaborating Centre on eHealth, School of Public Health and Community Medicine, Faculty of Medicine, University of New South Wales, Sydney, Australia

**Keywords:** Gene expression, Gene ontology, KEGG pathway analysis, PPI network, Molecular biomarkers

## Abstract

**Background:**

Worldwide, more than 80% of identified lung cancer cases are associated to the non-small cell lung cancer (NSCLC). We used microarray gene expression dataset GSE10245 to identify key biomarkers and associated pathways in NSCLC.

**Results:**

To collect Differentially Expressed Genes (DEGs) from the dataset GSE10245, we applied the R statistical language. Functional analysis was completed using the Database for Annotation Visualization and Integrated Discovery (DAVID) online repository. The DifferentialNet database was used to construct Protein–protein interaction (PPI) network and visualized it with the Cytoscape software. Using the Molecular Complex Detection (MCODE) method, we identify clusters from the constructed PPI network. Finally, survival analysis was performed to acquire the overall survival (OS) values of the key genes. One thousand eighty two DEGs were unveiled after applying statistical criterion. Functional analysis showed that overexpressed DEGs were greatly involved with epidermis development and keratinocyte differentiation; the under-expressed DEGs were principally associated with the positive regulation of nitric oxide biosynthetic process and signal transduction. The Kyoto Encyclopedia of Genes and Genomes (KEGG) pathway investigation explored that the overexpressed DEGs were highly involved with the cell cycle; the under-expressed DEGs were involved with cell adhesion molecules. The PPI network was constructed with 474 nodes and 2233 connections.

**Conclusions:**

Using the connectivity method, 12 genes were considered as hub genes. Survival analysis showed worse OS value for SFN, DSP, and PHGDH. Outcomes indicate that Stratifin may play a crucial role in the development of NSCLC.

## Background

Past few years, lung cancer was taking the leading role in cancer-related death. According to the study, in 2018, worldwide lung cancer was the most common cancer type by contributing 2.01 million diagnosed cases and approximately 1.8 million deaths [1]. Non-small-cell lung carcinoma (NSCLC) is the most common type of lung cancer; more than 80% of patients with lung cancer were affected by NSCLC [2].. Adenocarcinoma is the most ordinary type of lung cancer; approximately 40% of NSCLC is adenocarcinoma. This type of NSCLC arises from small airway epithelial, type II alveolar cells, which secrete mucus and other substances [3]. Smoking is listed as one of the worst risk factors for adenocarcinoma [4]. Squamous cell carcinoma (SCC) is the second most common subtype of all lung cancer cases; it constitutes 25–30% of all lung cancer. SCC is sharply correlated with smoking [5].

In recent years, the development in genomics, molecular biology, as well as DNA sequencing methods has guided the identification of many dynamic factors as molecular signature, which may provide better chances for the early detection of cancer [6]. Microarray terminology is referred to as a high-throughput platform used to analyze gene expression and has been broadly used to obtain gene alteration during tumorigenesis and identify prognostic biomarkers in patients with cancer [7, 8]. In this investigation, we aimed to identify molecular biomarkers for NSCLC using microarray technology, which may help its early diagnosis and prognosis.

**Fig. 1:**
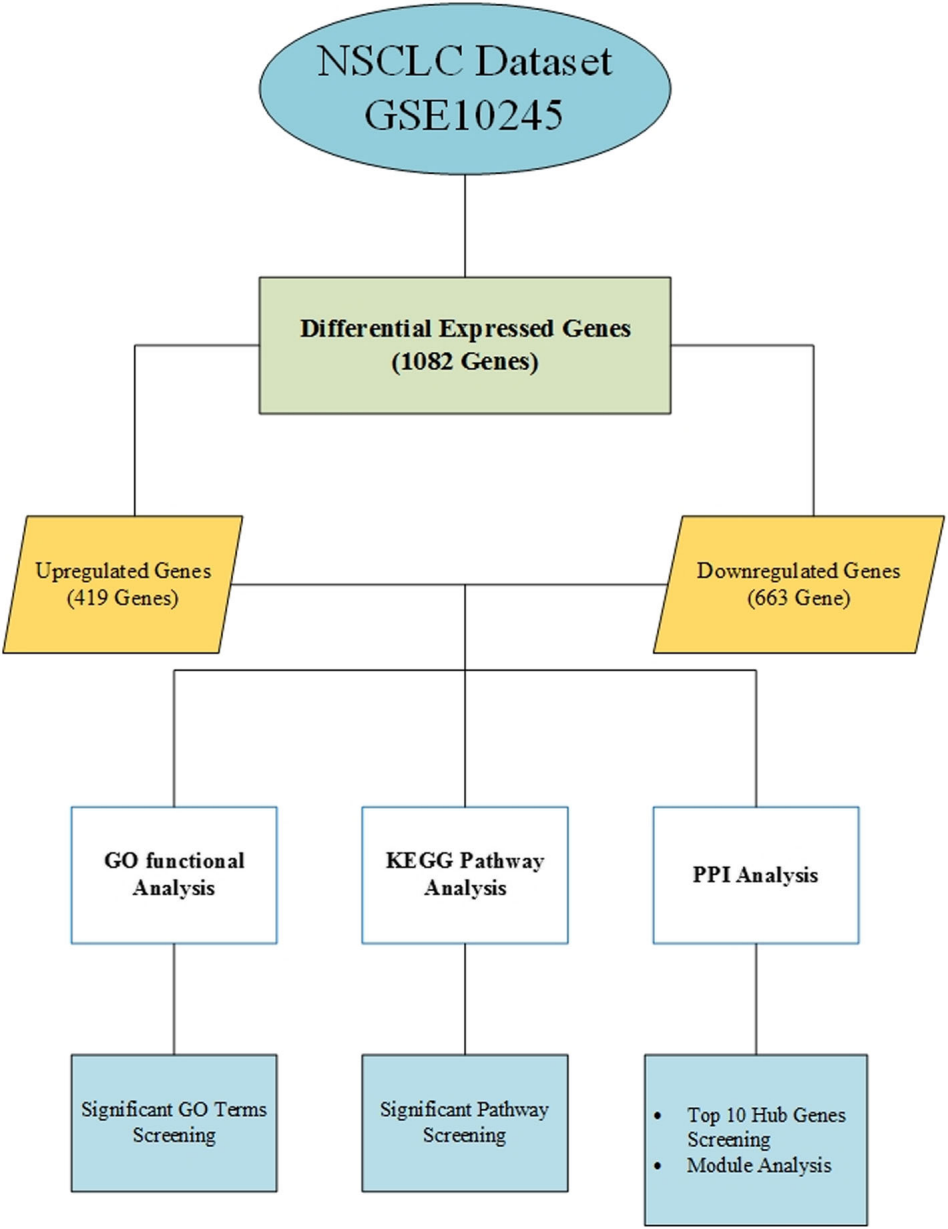
Flow diagram of the analytical approach used in this investigation

In this study, we collected microarray dataset GSE10245 from the Gene Expression Omnibus (GEO) database and utilized R language to identify the differentially expressed genes (DEGs) between adenocarcinoma and SCC. After identifying the DEGs, functional and pathway analysis was performed by using Database for Annotation, Visualization, and Integrated Discovery (DAVID) functional database. To predict the protein–protein interaction (PPI) network, we used the DifferentialNet repository. The PPI network was visualized by the Cytoscape tool. The Molecular Complex Detection (MCODE) technique was fruitful to perform module analysis from the constructed PPI network. We calculate connectivity degree value to identify hub genes. After that, the overall survival (OS) analysis was done by using the Kaplan–Meier (KM) plotter. The goal of this investigation is to identify molecular biomarkers, to make potential therapeutic medicine for future NSCLC treatment. Figure 1 shows the flow chart of the present study.

## Methods

### Gene expression profile data

Selected microarray gene expression profile GSE10245 was collected from the NCBI’s GEO (http://www.ncbi.nlm.nih.gov/geo/) repository [9, 10]. The GPL570 platform with Affymetrix Human Genome U133 Plus 2.0 Array was used for the dataset. Fifty-eight NSCLC-associated samples were found in GSE10245.

### Differentially expressed gene (DEG) screening

The selected profile GSE10245 was converted into expression measures using the Linear Models for Microarray and RNA-Seq Data (limma) of the R language [11]. Identified DEGs were collected following the cut-off criteria: |log fold-change (FC)|> 1.25 and *P* value < 0.05.

### Functional analysis of DEGs

In the present Bioinformatics analysis, Gene Ontology (GO) analysis is a widely used method to know functional annotation of a gene set [12]. The Kyoto Encyclopedia of Genes and Genomes (KEGG) database contains genomic information, recognized pathways, gene functions, and gene networks with higher-order functional information of various organisms [13]. The DAVID (http://david.ncifcrf.gov/) is an online tool that can provide wide functional information about genes/proteins [14]. The present study used the DAVID tool to identify important GO terms and KEGG pathways of identified DEGs.

### Protein–protein interaction and module analysis construction

DifferentialNet repository was used to foretell the potential interaction between gene products in the human lung tissue. The DifferentialNet is a great repository that supplies human organ tissue-specific interactomes information (http://netbio.bgu.ac.il/diffnet/) [15]. Twenty percent of filter interactions were considered as significant. The integration of protein–protein interaction (PPI) networks was constructed by using Cytoscape (Version 3.7.2) [16]. Degree > 5 was set as the cutoff criteria for the PPI networks. The MCODE algorithm was utilized to identify modules from the PPI network [17]. Additionally, MCODE score > 2 and amount of node > 10 were set as cutoff standard to perform the module analysis. After performing the module analysis, we used the DAVID functional database to perform the KEGG pathway analysis of top gene modules. Finally, we identified hub genes based on higher-degree connectivity value in the PPI network.

### Survival analysis of hub genes

The Kaplan–Meier (KM) plotter (http://kmplot.com/analysis/) online Bioinformatics tool that count the effect of more than 54,000 genes on survival by using around 11,000 samples, including 6234 breast cancer samples, 2190 ovarian cancer samples, 3452 lung cancer samples, and 1440 gastric cancer samples [18]. The overall survival analysis-related information was based on the European Genome-Phenome Archive (EGA), GEO, and The Cancer Genome Atlas (TCGA) database. In the KM plotter, the hazard ratio (HR) and low-rank *P* value were considered and showed on the plot.

## Results

### Differentially expressed genes (DEG) screening

The GSE10245 gene expression profile was elected in this study. The selected gene expression profile had a total of 58 samples, including 40 ADC samples and 18 SCC samples. Based on criteria |log (FC)|> 1.25 and *P* value < 0.05, a total of 1082 DEGs were identified from the analyzed dataset, including 419 DEGs were overexpressed and 663 DEGs were under-expressed.

### Functional analysis of DEGs

The GO function terms for DEGs were identified by using the DAVID online database. The overexpressed genes were significantly enhanced in the function of epidermis development, mitotic nuclear division, and keratinocyte differentiation for Biological Process (BP), chromosome, centromeric region and cytoplasm for Cellular Component (CC), and structural molecule activity and microtubule binding for Molecular Function (MF) (Table 1). The under-expressed genes were significantly enhanced in the functions of positive regulation of nitric oxide biosynthetic process and signal transduction for BP, extracellular exosome and extracellular space for CC, and scavenger receptor activity and growth factor activity for MF (Table 2).

**Table 1:** The functional analyses of upregulated genes to identify top 15 GO terms

Category	Term name	Count	***P*** value
BP	GO:0008544—epidermis development	20	2.13E–14
BP	GO:0030216—keratinocyte differentiation	18	5.06E–13
BP	GO:0030855—epithelial cell differentiation	11	2.79E–06
BP	GO:0031424—keratinization	9	8.65E–06
BP	GO:0007067—mitotic nuclear division	19	9.58E–06
CC	GO:0001533—cornified envelope	10	4.31E–07
CC	GO:0030057—desmosome	7	8.46E–06
CC	GO:0000775—chromosome, centromeric region	9	2.51E–05
CC	GO:0005737—cytoplasm	148	3.40E–05
CC	GO:0005882—intermediate filament	11	1.45E–04
MF	GO:0005198—structural molecule activity	24	3.60E–09
MF	GO:0008017—microtubule binding	15	1.60E–04
MF	GO:0001758—retinal dehydrogenase activity	4	3.18E–04
MF	GO:0005200—structural constituent of cytoskeleton	10	5.89E–04
MF	GO:0042803—protein homodimerization activity	30	0.001048

*BP* biological process, *CC* cellular component, *MF* molecular function

**Table 2:** The functional analyses of downregulated genes to identify top 15 GO terms

Category	Term name	Count	***P*** value
CC	GO:0070062—extracellular exosome	170	1.57E–14
CC	GO:0005615—extracellular space	97	3.11E–12
CC	GO:0005886—plasma membrane	200	2.84E–08
CC	GO:0005887—integral component of plasma membrane	86	2.00E–07
CC	GO:0005576—extracellular region	93	5.62E–07
BP	GO:0045429—positive regulation of nitric oxide biosynthetic process	9	1.02E–04
BP	GO:0007165—signal transduction	65	1.72E–04
BP	GO:0050714—positive regulation of protein secretion	8	2.08E–04
BP	GO:0005975—carbohydrate metabolic process	17	3.93E–04
BP	GO:0050873—brown fat cell differentiation	7	7.29E–04
MF	GO:0005044—scavenger receptor activity	8	0.001241
MF	GO:0008083—growth factor activity	14	0.004251
MF	GO:0042803—protein homodimerization activity	40	0.005049
MF	GO:0005088—Ras guanyl-nucleotide exchange factor activity	11	0.00664
MF	GO:0003779—actin binding	19	0.008431

In addition, KEGG pathway analyses for the overexpressed and under-expressed DEGs were accomplished using the DAVID database. The overexpressed DEGs were momentously enhanced in chemical carcinogenesis, cell cycle, and Hippo signaling pathway (Table 3), while the under-expressed DEGs were greatly enhanced in complement and coagulation cascades, cell adhesion molecules, and tight junction (Table 4).

**Table 3:** The pathway analyses of upregulated genes

Term ID	Term name	Count	***P*** value
hsa00980	Metabolism of xenobiotics by cytochrome P450	12	1.02E–06
hsa00982	Drug metabolism—cytochrome P450	9	1.77E–04
hsa05204	Chemical carcinogenesis	9	5.47E–04
hsa04110	Cell cycle	11	6.45E–04
hsa04390	Hippo signaling pathway	12	8.23E–04
hsa00480	Glutathione metabolism	7	0.00117
hsa04550	Signaling pathways regulating pluripotency of stem cells	9	0.01722
hsa04514	Cell adhesion molecules	9	0.0186
hsa04115	p53 signaling pathway	6	0.02035

**Table 4:** The pathway analyses of downregulated genes

Term ID	Term name	Count	***P*** value
hsa04610	Complement and coagulation cascades	14	1.21E–06
hsa04514	Cell adhesion molecules	16	2.49E–04
hsa05150	Staphylococcus aureus infection	8	0.003691
hsa04530	Tight junction	10	0.004956
hsa04974	Protein digestion and absorption	10	0.005345
hsa05414	Dilated cardiomyopathy	9	0.01278
hsa04950	Maturity onset diabetes of the young	5	0.014881
hsa01100	Metabolic pathways	59	0.025714
hsa00220	Arginine biosynthesis	4	0.036721

### PPI and module analysis

**Fig. 2:**
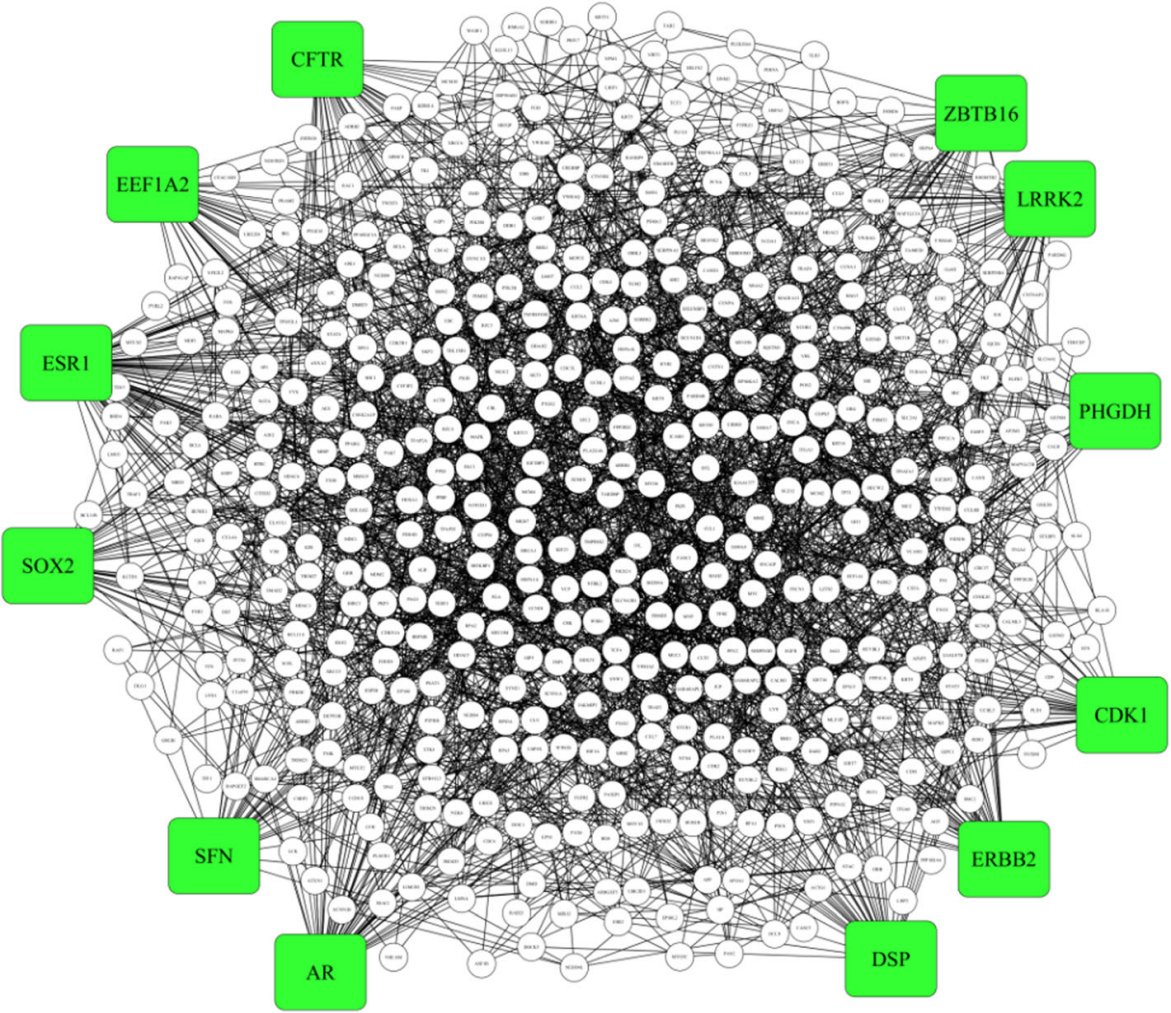
Protein–protein interaction network with 474 nodes and 2233 connections. Highlighted green color nodes indicate Hub genes of the network

Protein interactions of lung tissue among the identified 1082 DEGs were predicted with the DiffentialNet database. Four hundred seventy four nodes with 2233 connections were attached in the constructed PPI network as showed in Fig. 2. In our PPI analysis, we consider the connectivity degree value method to identify hub genes. Connectivity degree values of more than 34 were considered hub genes (Table 5). Outcomes from the PPI network revealed that Estrogen Receptor 1 (ESR1) was the most eminent gene with the highest connectivity degree value (80), followed by AR (degree value = 51), LRRK2 (degree value = 45), CFTR (degree value = 40), DSP (degree value = 39), ZBTB16 (degree value = 39), ERBB2 (degree value = 38), CDK1 (degree value = 36), EEF1A2 (degree value = 36), PHGDH (degree value = 35), SFN (degree value = 35), and SOX2 (degree value = 35). There were 5 overexpressed and 7 under-expressed genes in identified 12 hub genes.

**Table 5:** List of hub genes and value of the degree of connectivity

Gene symbol	Gene name	Degree of connectivity
ESR1	Estrogen receptor 1	80
AR	Androgen receptor	51
LRRK2	Leucine-rich repeat kinase 2	45
CFTR	CF transmembrane conductance regulator	40
ZBTB16	Zinc finger and BTB domain-containing 16	39
ERBB2	Erb-B2 receptor tyrosine kinase 2	38
EEF1A2	Eukaryotic translation elongation factor 1 alpha 2	36
DSP	Desmoplakin	39
CDK1	Cyclin-dependent kinase 1	36
PHGDH	Phosphoglycerate dehydrogenase	35
SFN	Stratifin	35
SOX2	SRY-box transcription factor 2	35

**Fig. 3:**
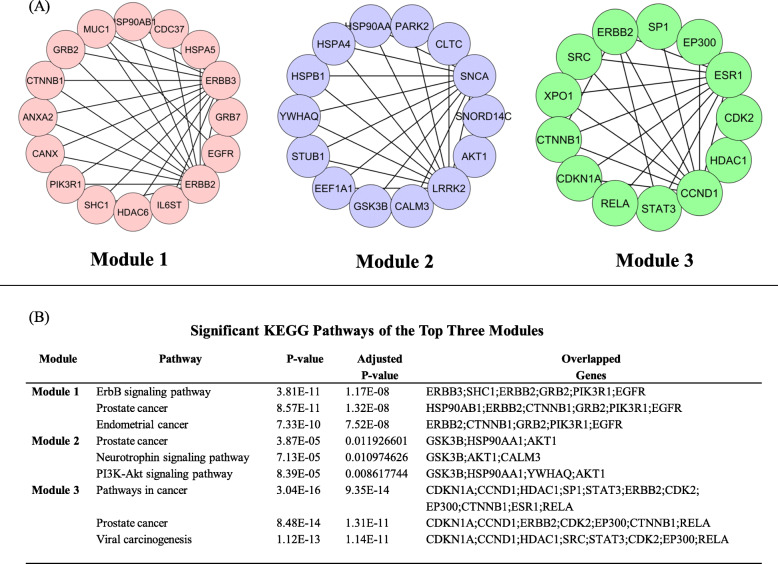
**a** Top 3 module analysis of PPI network. **b** Module-associated top significant pathways

In this study, the MCODE algorithm was used to identify significant modules by analyzing the constructed PPI network. Thirty one clusters were found using the MCODE algorithm; we identify the top 3 clusters among them (Fig. 3a). The pathway analysis explored that the three modules were principally connected with ErbB signaling pathway, Prostate cancer, and Viral carcinogenesis (Fig. 3b).

### Survival analysis of hub genes

**Fig. 4:**
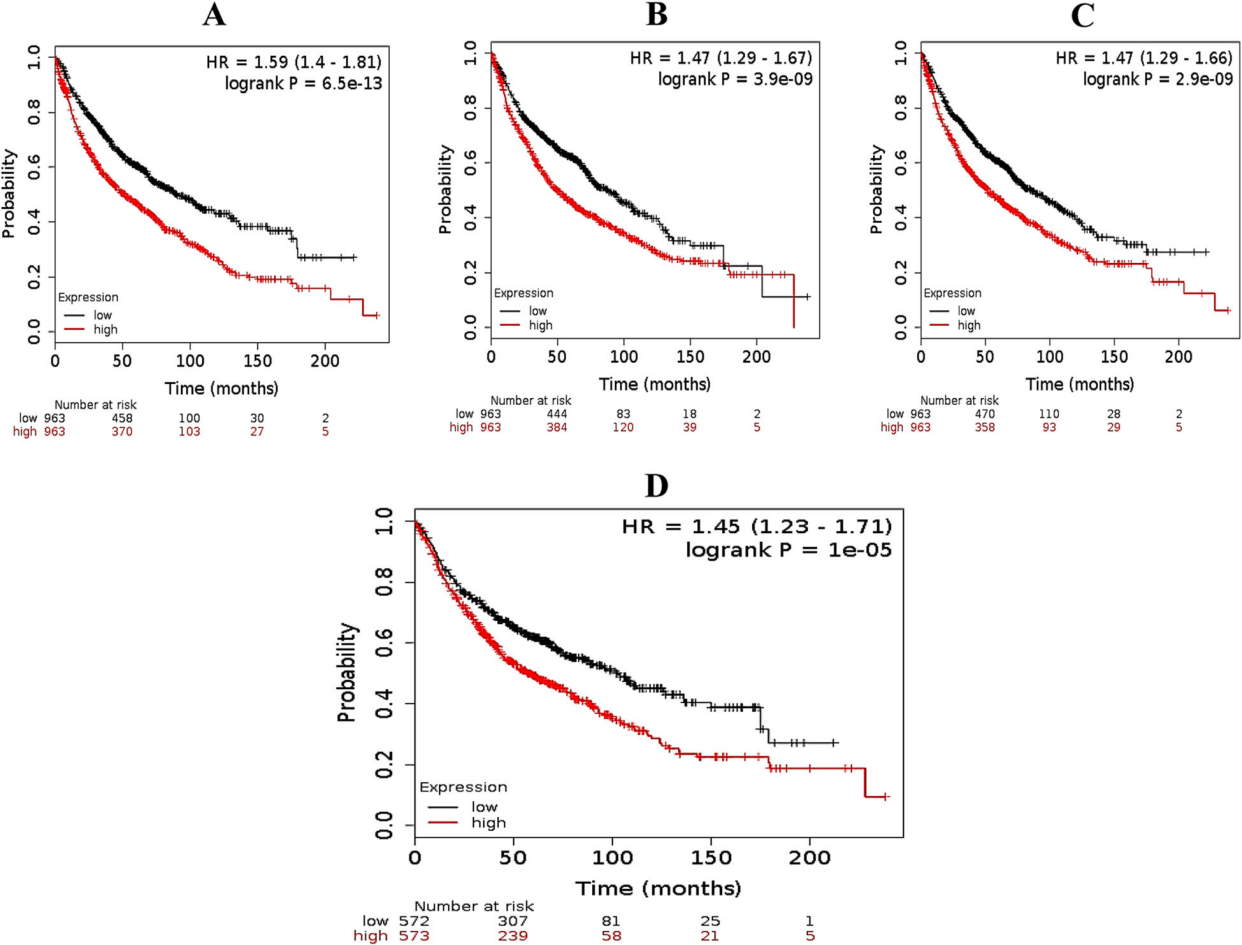
Overall survival analysis diagram. **a** SFN, HR = 1.59; **b** DSP, HR = 1.47; **c** PHGDH, HR = 1.47; **d** overall mean of hub genes, HR = 1.45

The KM plotter online experiment tool was used to observe the prognostic values of the identified hub genes. A total of 1926 patient’s records were available for the overall survival (OS) analysis. The KM plotter analysis shows that the expression of SFN (HR = 1.59 [1.4–1.81], low-rank *P* = 6.5e–13) (Fig. 4a) was engaged with worse OS for lung cancer patients, as well as DSP (HR = 1.47 [1.29–1.67], low-rank *P* = 3.9e–09) (Fig. 4b) and PHGDH (HR = 1.47 [1.29–1.66], low-rank *P* = 2.9e–09) (Fig. 4c) and identified 12 hub genes mean OS [HR = 1.45 [1.23–1.71], low-rank *P* = 1e–05] (Fig. 4d).

## Discussion

NSCLC has been a broadly studied topic in cancer research, though there is still a shortage of early detection and diagnosis. Generally, symptoms of NSCLC do not become evident until the cancer is already at an advanced stage; this is one of the principal causes of lacking early detection and diagnosis of NSCLC. Bioinformatics analysis has rapidly increased in the last few years for discovering new therapeutic targets and biomarkers for several cancers [19]. In 2020, Maharjan et al., using bioinformatics analysis, identified 16 biomarkers for lung cancer including Cyclin-B2 (CCNB2), Cell Division Cycle 20 (CDC20), F-Box And Leucine Rich Repeat Protein 3 (FBXL3), and Forkhead Box A2 (FOXA2) [20]. Dai et al. identified CDC20, ECT2, MKI67, TPX2, and TYMS as biomarkers using microarray analysis, where Cell Division Cycle 20 (CDC20), Epithelial Cell Transforming 2 (ECT2), Marker of Proliferation Ki-67 (MKI67), TPX2 Microtubule Nucleation Factor (TPX2), and Thymidylate Synthetase (TYMS) showed worse survival outcome [21]. Few studies reveal that Cyclin A2 (CCNA2) and Neuromedin U (NMU) were involved with diagnosis and prognosis of NSCLC [22, 23].

In the current study, 1082 DEGs were collected from gene expression dataset GSE10245, including 419 overexpressed DEGs and 663 under-expressed DEGs. The 419 overexpressed genes were significantly enhanced in the function of epidermis development and keratinocyte differentiation for Biological Process (BP), and the 663 downregulated genes were significantly enhanced in the functions of positive regulation of nitric oxide biosynthetic process and signal transduction for BP. The KEGG pathways analysis explored that the overexpressed DEGs were momentously enhanced in Chemical carcinogenesis, Cell cycle, and Hippo signaling pathway, while the under-expressed DEGs were momentously enhanced in Complement and coagulation cascades, Cell adhesion molecules, and Tight junction. The PPI network was constructed with 474 genes and 2233 connections. Twelve genes were deliberated as hub genes including ESR1, AR, LRRK2, CFTR, ZBTB16, DSP, ERBB2, EEF1A2, CDK1, PHGDH, SFN, and SOX2. The top three modules were mainly associated with the ErbB signaling pathway, Prostate cancer, and Viral carcinogenesis. Kaplan–Meier plotter showed that the high expression of 3 out of 12 hub genes was attached with worse OS value, including SFN, DSP, and PHGDH.

Stratifin (SFN) is a member of the 14-3-3 protein family, a highly preserved group of proteins participating by 7 isoforms. SFN is engaged with many significant biological functions like cell cycle apoptosis, regulation of signal transduction pathways, and cell proliferation [24, 25].. SFN often plays a role in inhibiting DNA errors during mitosis to respond to DNA damage [26]. SFN had a high expression of malignant progression in early-stage lung adenocarcinoma [24, 27]. Besides, associated with OCIAD2, immunocytochemical staining for SFN could also increase diagnostic sensitivity for lung cancers [28]. SFN gene expression was notably increased and displayed high protein expression in immunohistochemical tarnish of TP53 mutated tumors [29]. In addition, previous study reported that SFN gets involved with multiple kinds of tumor progression including breast, liver, ovarian, and renal tumors [30]. SFN shows also poor OS value in our survival analysis. SFN may play a vital role in the progression of NSCLC. Estrogen receptor 1 (ESR1) gene plays an active role in the progression of various cancers such as breast, prostate, and endometrial cancer [31–33]. ESR1 gene plays an active role in metastatic breast cancer [34, 35]. Previous report revealed that estrogen receptors (ERs) play significant role in NSCLC progression [36]. ERs might influence several cancer-associated biological functions and pathways in NSCLC, notably, membrane receptor activation and signal transduction, which might ultimately lead the way to changes in cell behaviors. In a recent study, Xiujuan Gao shows that ERs help to develop NSCLC by modulating the membrane receptor signaling network [37]. So ESR1 also may play an active role in the development of NSCLC. Cyclin-dependent kinase 1 (CDK1) covers a vital role in the monitoring of the cell cycle by regulating the centrosome cycle. CDK1 serves as a prognostic biomarker for cancers including colorectal and lung cancers [38, 39]. Desmoplakin (DSP) is an originating member of the plakin family; DSP is a committed element of desmosomal plaques [40]. Yang et al. showed that DSP acts as a tumor suppressor in lung cancer [41]. The limitations of our study were as follows. First, we use only one dataset. Second, the sample size of the dataset was comparatively small. Third, we could not validate due to the absence of experiments. But we hope our study will make a positive impact to identify biomarkers of NSCLC.

## Conclusion

In summary, we analyzed a microarray dataset GSE10245 of NSCLC and identified 1082 DEGs including 419 upregulated and 663 downregulated DEGs that connected with NSCLC. Functional enrichment analysis explored that overexpressed DEGs were greatly involved with epidermis development and keratinocyte differentiation; the under-expressed DEGs were principally associated with the positive regulation of nitric oxide biosynthetic process and signal transduction. The KEGG pathway analysis showed that the overexpressed DEGs were highly involved with the cell cycle; and the under-expressed DEGs were involved with cell adhesion molecules. From the PPI network analysis, we have found 12 hub genes which has more than or equal 35 connections in the network. After implementing the MCODE method, 3 significant clusters were detected, the clusters were mainly connected with ErbB signaling pathway, Prostate cancer, and Viral carcinogenesis. Survival analysis explored that SFN had the worst HR value. Depending on our investigation, we can say that Stratifin (SFN) may play as a biomarker in the progression of NSCLC. Further study needed to confirm our statement.

Acknowledgements

This manuscript has not been published yet and not even under consideration for publication elsewhere. The authors are grateful who have participated in this research work. We thank the anonymous referees for their useful suggestions.

## Data Availability

The datasets used and/or analyzed during the current study are available from the corresponding author on reasonable request.

## Abbreviations

NSCLC: Non-small-cell lung carcinoma
DEGs: Differentially expressed genes
GEO: Gene Expression Omnibus
NCBI: National Center of Biotechnology Information
KEGG: Kyoto Encyclopedia of Genes and Genomes
GO: Gene Ontology
DAVID: Database for Annotation, Visualization, and Integrated Discovery
KM plotter: Kaplan–Meier plotter
MCODE: Molecular Complex Detection
Limma: Linear Models for Microarray and RNA-Seq Data
